# Functional Significance of AtHMA4 C-Terminal Domain *In Planta*

**DOI:** 10.1371/journal.pone.0013388

**Published:** 2010-10-20

**Authors:** Rebecca F. Mills, Billy Valdes, Michael Duke, Kerry A. Peaston, Brett Lahner, David E. Salt, Lorraine E. Williams

**Affiliations:** 1 School of Biological Sciences, University of Southampton, Southampton, Hampshire, United Kingdom; 2 Center for Plant Environmental Stress Physiology, Purdue University, West Lafayette, Illinois, United States of America; University of Massachusetts Amherst, United States of America

## Abstract

**Background:**

Enhancing the upward translocation of heavy metals such as Zn from root to shoot through genetic engineering has potential for biofortification and phytoremediation. This study examined the contribution of the heavy metal-transporting ATPase, AtHMA4, to the shoot ionomic profile of soil-grown plants, and investigated the importance of the C-terminal domain in the functioning of this transporter.

**Principal Findings:**

The *Arabidopsis hma2 hma4* mutant has a stunted phenotype and a distinctive ionomic profile, with low shoot levels of Zn, Cd, Co, K and Rb, and high shoot Cu. Expression of *AtHMA4* (*AtHMA4-FL*) under the CaMV-35S promoter partially rescued the stunted phenotype of *hma2 hma4*; rosette diameter returned to wild-type levels in the majority of lines and bolts were also produced, although the average bolt height was not restored completely. *AtHMA4-FL* expression rescued Co, K, Rb and Cu to wild-type levels, and partially returned Cd and Zn levels (83% and 28% of wild type respectively). In contrast, expression of *AtHMA4-trunc* (without the C-terminal region) in *hma2 hma4* only partially restored the rosette diameter in two of five lines and bolt production was not rescued. There was no significant effect on the shoot ionomic profile, apart from Cd, which was increased to 41% of wild-type levels. When the *AtHMA4* C-terminal domain (*AtHMA4-C-term*) was expressed in *hma2 hma4* it had no marked effect. When expressed in yeast, *AtHMA4-C-term* and *AtHMA4-trunc* conferred greater Cd and Zn tolerance than *AtHMA4-FL*.

**Conclusion:**

The ionome of the *hma2 hma4* mutant differs markedly from wt plants. The functional relevance of domains of AtHMA4 *in planta* can be explored by complementing this mutant. AtHMA4-FL is more effective in restoring shoot metal accumulation in this mutant than a C-terminally truncated version of the pump, indicating that the C-terminal domain is important in the functioning of AtHMA4 *in planta*.

## Introduction

Zn is an essential element with diverse roles in biological systems. It is increasingly recognized as being of the utmost importance for human health and quality of life and is an essential dietary element [1]. Zn deficiency in humans is widespread with an estimated 30% of the world’s population at risk [2]. Extreme cases of Zn deficiency result in impaired infant growth and development [3] and there is now strong evidence that even mild Zn deficiency contributes significantly to the many deaths annually worldwide from malaria, diarrhoea, measles and pneumonia that have been attributed to malnutrition [4]. Zn supplementation improves child growth and decreases child mortality [5] and dietary Zn supplementation reduces the prevalence of infectious disease in populations at risk of Zn deficiency [6]. The ultimate goal of modern agriculture is to produce nutritious and safe foods in sufficient quantities and in a sustainable manner. Biofortification is the process of increasing the natural content of bioavailable nutrients in plants while the plant is still growing, as opposed to post-harvest fortification. This allows the nutrient enrichment to be cost-effective and targeted, particularly if performed using genetic approaches. Producing Zn-enriched plant food products by such methods would potentially generate major health benefits [7]. Furthermore, plants yield less and have a lower nutritional quality when grown in soils where Zn availability is low [8], [9]. Therefore the development of Zn-efficient plants (plants that can maintain growth and yields under low soil Zn) would have clear benefits for agriculture [7].

To optimise crop improvements it is important to have a clear understanding of Zn transport and homeostasis in plants. Several key families of transporters have been shown to have a role in this and are therefore potential targets for use in biofortification strategies. The P_1B_-ATPase family plays an important role in heavy metal transport in plants. There are eight P_1B_-ATPases in *Arabidopsis thaliana* and four of these have been shown to have some role in Zn transport. AtHMA4 was the first member of the Zn/Cd/Pb/Co subclass of plant P_1B_-ATPases to be functionally characterized [10] and studies in yeast provided evidence that this heavy metal ATPase can transport Zn and the toxic element Cd [10], [11]. This pump and the related P_1B_-ATPase, HMA2, are essential for efficient translocation of Zn from roots to shoots in *A. thaliana* [12], [13] but they are also a route for Cd transport [10]–[15].

AtHMA4 has eight predicted transmembrane domains with a cytoplasmic loop between transmembrane domains 4 and 5, and a larger loop between transmembrane domains 6 and 7. It is also predicted to possess a short cytoplasmic domain at the N-terminus and a long cytoplasmic domain at the C-terminus (extending approximately 470 amino acids after the end of the last predicted transmembrane domain) that may play regulatory roles [11]. Studies in yeast showed that a truncated version of AtHMA4, Athma4Δ714–1172 (lacking the cytoplasmic C-terminal region) conferred greater Zn tolerance than full-length AtHMA4 to the Zn-sensitive mutant *zrc1 cot1* when subjected to elevated Zn and greater Cd tolerance to wt yeast [11]. The C-terminal region expressed alone in yeast also conferred tolerance to Cd [16].

Previously it has been reported that over-expression of *AtHMA4* in the Ws ecotype of *Arabidopsis* driven by the cauliflower mosaic virus 35S promoter can enhance tolerance to high levels of Zn, Cd and Co [13]. This correlated with an increase in Cd and Zn in the leaves (root levels did not change significantly) [13]. Ectopic expression of *AtHMA4* in tobacco results in responses that differ with external Zn or Cd concentrations, highlighting the importance of endogenous homeostatic interactions [17]. In the Zn hyperaccumulator *Arabidopsis halleri,* Zn and Cd hypertolerance depend on the AtHMA4 homologue, AhHMA4. In this species, gene copy number triplication combined with enhanced expression of the three *A. halleri HMA4* genes are thought to have been important in the evolution of hypertolerance [18]. Expressing *AhHMA4* under the 35S promoter in *Arabidopsis thaliana* also resulted in enhanced tolerance to elevated levels of Zn and Cd; however shoot concentrations in these plants was unchanged or reduced by around 35–45%, suggesting that in these plants Zn and Cd tolerance was conferred by exclusion [18].

In *Arabidopsis thaliana*, HMA2 and HMA4 are expressed in the vascular system and are important for the translocation of Zn and Cd from roots to shoots [12]–[14]. Whereas neither the *hma2* nor the *hma4* mutant has an easily visible growth phenotype, the *hma2 hma4* double mutant is severely stunted (even in the Ws mutant that retains a full-length version of HMA3), and this phenotype is rescued by application of Zn [12]. Closer inspection reveals that the *hma4* mutant has slightly reduced seed and silique size [11].

Recently it was shown that *AtHMA2* expressed under its own promoter was able to rescue the Zn deficiency phenotype and Cd transport defect of the *hma2 hma4* mutant to levels observed in the single *hma4* mutant [15]. Deletion of the N-terminal 74 amino acids of HMA2 abolished this ability [15] but deletion of the C-terminal cytoplasmic domain had little effect on the rescue. C-terminal truncated versions of *AtHMA2* still rescued the Zn deficiency stunted phenotype of the *hma2 hma4* mutant, although the version with the entire C-terminal region removed barely rescued the sterility phenotype [15]. The cytoplasmic C-terminal domains of HMA2 and HMA4 contain potential metal-binding motifs including multiple interspersed Cys pairs and His residues in various motifs. The C-terminal domain of HMA2 has been shown to bind three Zn^2+^ ions with high affinity [19].

Ionomics is the study of an organism’s elemental composition using high throughput technologies. In this study we analysed the shoot ionome of the *hma2 hma4* double mutant together with the *hma2* and *hma4* single mutants. We tested the ability of full length *AtHMA4* and two partial versions of *AtHMA4* expressed from the 35S promoter, to rescue the Zn deficiency phenotype of the Arabidopsis *hma2 hma4* double mutant, and to restore the defect in the ability of this mutant to accumulate Cd in shoots. In particular, the aim was to determine whether C-terminal truncation of AtHMA4 had any effect on its function in *Arabidopsis* and to determine whether the HMA4 versions had potential in future biofortification strategies.

## Results

Three different *AtHMA4* constructs were investigated in this study: full-length *AtHMA4* (*AtHMA4-FL*); Athma4Δ714-1172 with the C-terminal 459 amino acids of AtHMA4 removed (*AtHMA4-trun*c); Athma4Δ1–699, comprising only the C-terminal 473 amino acids of AtHMA4 (*AtHMA4-C-term*) (see Figure S1). In addition, two point mutation constructs (D_401_A and C_357_G) were included as transport null mutants for the yeast complementation analyses (Figure S1).

### Tolerance conferred to yeast by AtHMA4 variants

**Figure 1:**
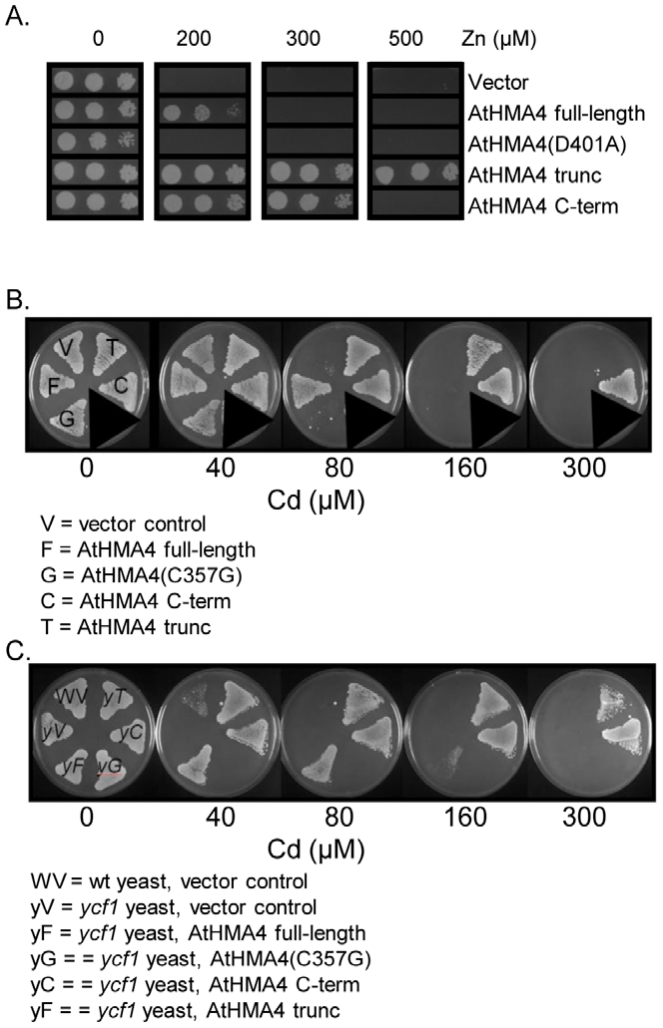
Direct comparison of Zn and Cd tolerance conferred by AtHMA4 and truncated versions in yeast. *AtHMA4-FL*, *AtHMA4-trunc* and *AtHMA4-C-term* were expressed in *zrc1 cot1* yeast mutant (A), wt yeast (BY4741) (B) or *ycf1* mutant (C). Growth of yeast expressing these AtHMA4 versions were compared to vector (p426)-transformed controls and to either of two transport null mutants: Athma4(D401A) or AtHMA4(C357G). Plates contained SC minus uracil with 2% (w/v) galactose pH 5-5.5 and varying concentrations of Cd as CdSO_4_ or Zn as ZnSO_4_.

*AtHMA4-F*L, *AtHMA4-trunc* and *AtHMA4-C-term* were expressed in various yeast strains to determine their relative effectiveness in conferring Zn and Cd tolerance. We also included either of two transport null mutants: Athma4(D401A), mutated in the conserved aspartate phosphorylated during the reaction cycle of all P-type ATPases or Athma4(C357G), mutated in the conserved CPC motif [11]. In these experiments, yeast were grown at pH 5–5.5 on a minimal medium with galactose to induce expression. Consistent with previous studies, full-length AtHMA4 confers Zn tolerance to the Zn-sensitive *zrc1 cot1* mutant yeast when grown under these conditions (Figure 1A) and deletion of the C-terminal 459 amino acids results in greater Zn tolerance [11]. No tolerance is observed when the transport null Athma4(D401A) mutant is expressed (Figure 1A). Here we show that expression of the 473 amino acid C-terminus alone (*AtHMA4-C-term*) conferred greater Zn tolerance to *zrc1 cot1* yeast than *AtHMA4-FL*, although the tolerance was not as great as that conferred by *AtHMA4-trunc* (Figure 1A). Also demonstrated here are the relative abilities of the AtHMA4 variants in conferring Cd tolerance (Figure 1B and 1C). *AtHMA4-FL* expressed in wild-type yeast, confers Cd tolerance while the Athma4(C357G) mutant does not. AtHMA4-trunc conferred greater Cd tolerance than AtHMA4-FL whereas AtHMA4-C-term confers the greatest Cd tolerance (Figure 1B). In the *ycf1* mutant, as shown previously expression of *AtHMA4-FL* confers Cd tolerance [11], [20] and in this study (Figure 1C) we show that the truncated version confers greater tolerance. The difference between the C-term and truncated versions is not as clear but the AtHMA4-C-term appears slightly more tolerant at the highest concentration tested.

### Growth phenotype of the *A. thaliana hma2 hma4* mutant expressing versions of *AtHMA4* under the 35S promoter

From expression studies in yeast we found that while AtHMA4-FL confers tolerance to Zn and Cd, both the truncated form of the ion pump (lacking the C-terminal region) and the C-terminal region expressed alone conferred greater tolerance to these metals. In order to assess the function of these three constructs *in planta*, we expressed them in the *A. thaliana hma2 hma4* double knockout mutant [12] to observe their effect on the Zn deficiency phenotype.

**Figure 2:**
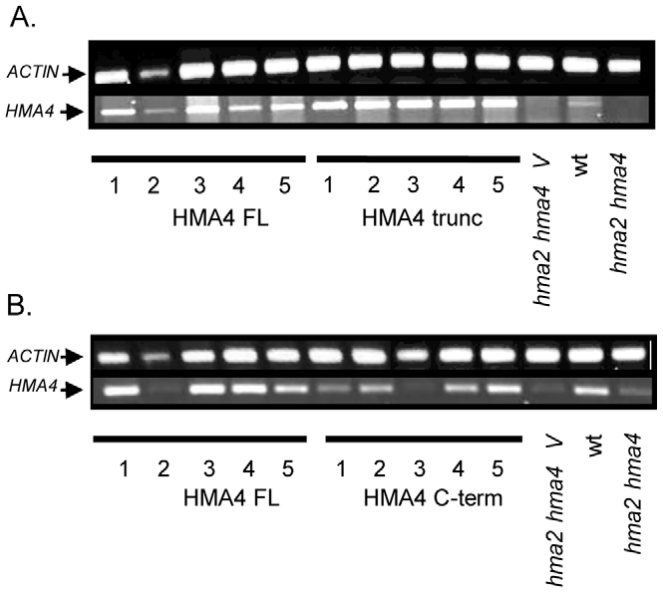
Arabidopsis *hma2 hma4* plants are expressing *AtHMA4-FL, AtHMA4-trunc* or *AtHMA4-C-term*. Semi-quantitative RT-PCR shows expression of *AtHMA4* versions in 5 independent transformant lines for each construct in the Arabidopsis *hma2 hma4* mutant. A, RT-PCR for lines expressing *AtHMA4 FL* (plants 1–5) or *AtHMA4-trunc* (plants 1–5) using primers that detect a region before the C-terminus.. B, RT-PCR for lines expressing *AtHMA4 FL* (plants 1–5) or *AtHMA4-C-term* (plants 1–5) using primers that detect a region within the C-terminus. Wild type plant (wt) and *hma2 hma4* mutant are shown as well as *hma2 hma4* mutant expressing vector alone (*hma2 hma4 V*). Actin was used as a control.

The *hma2 hma4* double knockout mutant was transformed with *AtHMA4-F*L, *AtHMA4-trunc* or *AtHMA4-C-term* expressed under control of the 35S promoter. We used RT-PCR to confirm disruption of the endogenous genes, and expression of the introduced constructs (Figure 2). One pair of primers was used to amplify a region in the first half of the *AtHMA4* cDNA (see Figure S2 for primer positions). These primers do not amplify any product from *hma2 hma4* or *hma2 hma4* vector control cDNA, showing that full length *AtHMA4* is not expressed in these plants. They amplify a product of the predicted size from cDNA of the *hma2 hma4* double knockout transformed either with the *35S-AtHMA4-FL* or the *35S-AtHMA4-trun*c construct (Figure 2A). This shows that *AtHMA4-FL* or *AtHMA4-trunc* constructs are being expressed in these *hma2 hma4* transformants. A second pair of primers was used to amplify *AtHMA4* cDNA within the region corresponding to the C-terminus of the protein. These primers amplified a product of the predicted size from wt *A. thaliana* and from the *hma2 hma4* double knockout transformed with *35S-AtHMA4-FL* or *35S-AtHMA4-C-term* (Figure 2B). A faint product from this region was also detected in *hma2 hma4* and *hma2 hma4* transformed with the empty vector, indicating low expression of a partial *AtHMA4* transcript from this mutant (Figure 2B). The T-DNA insertion in this gene occurs after the fourth transmembrane domain, in the cytoplasmic ‘A’ domain (see Figures S1 and S2 for insertion position). A partial transcript could be initiated downstream of the insertion. This would not be predicted to have any transport activity however it could mean that there are very low levels of the C-terminal region expressed in these plants which could have an effect on metal chelation. Overall the results show that the constructs are being expressed in these lines, but expression levels vary slightly between lines. AtHMA4 FL line 2 showed low amplification levels for *AtHMA4* but actin was also low in this sample. If the level of actin is taken into account then this line has similar expression levels to the other lines. AtHMA4-C-term line 3 also did not seem to show enhanced expression as the amplified product was comparable to that seen in the *hma2 hma4* mutant (Figure 2B). In this case actin levels were only slightly lower.

**Figure 3:**
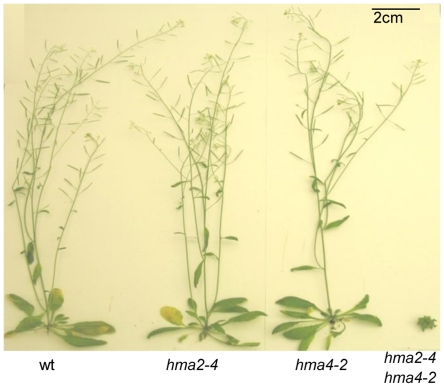
Comparison of wt and mutant plants grown on soil. Plants were grown for 42 days on soil under identical conditions in a controlled-environment growth room (22°C 16 h light, 20°C 8 h dark cycle). *Arabidopsis thaliana* (Columbia) wt, *hma2-4 (*SALK_034393), *hma4-2* (SALK_050924) and the double *hma2-4 hma4-2* mutant are shown.

Under the soil and growth conditions used in this study neither the *hma2-4* nor *hma4-2* single mutants showed any distinct vegetative growth phenotype, however the *hma2-4 hma4-2* double mutant was significantly stunted compared to wild-type (Figure 3). This mutant occasionally produced a bolt over the time frame of these experiments (45 days) but it was always very short. In contrast, at this stage wild type plants had bolted and produced flowers, siliques and seeds (Figure 3). After 60 days growth on soil, a small proportion of *hma2 hma4* mutants had produced several small bolts and some flowers, but siliques were not produced and thus no seed could be obtained. This was similar to the phenotype already reported for *hma2 hma4* in the Ws background [12] except that we did not observe that the plants in our study were chlorotic under our soil conditions as has been observed previously.

**Figure 4:**
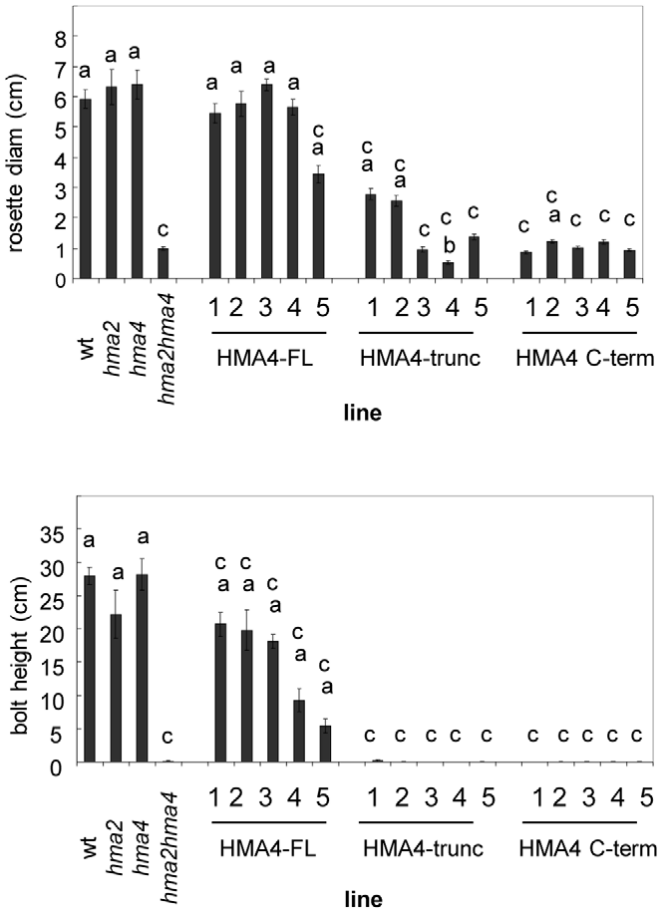
Rosette and bolt heights of lines expressing *AtHMA4-FL*, *AtHMA4-trun*c or *AtHMA4-C-term*. Growth of *AtHMA4*-expressing *hma2 hma4* plants (AtHMA4-FL, AtHMA4-trunc, AtHMA4-C-term) compared to untransformed *hma2 hma4* controls. Wild-type (wt) and *hma2* and *hma4* mutants are also shown. Plants were grown on soil under identical conditions in controlled-environment growth room (22°C 16 h light, 20°C 8 h dark cycle).Top, Rosette diameter and bottom, bolt height (42 days). Values are means +/− S.E. from at least 24 plants). Student’s t-test was used to determine significance levels. a  =  significantly larger than *hma2 hma4* (P<0.05), b =  line significantly smaller than *hma2 hma4* (P<0.05), c =  line significantly smaller than wt (P<0.05).

**Figure 5:**
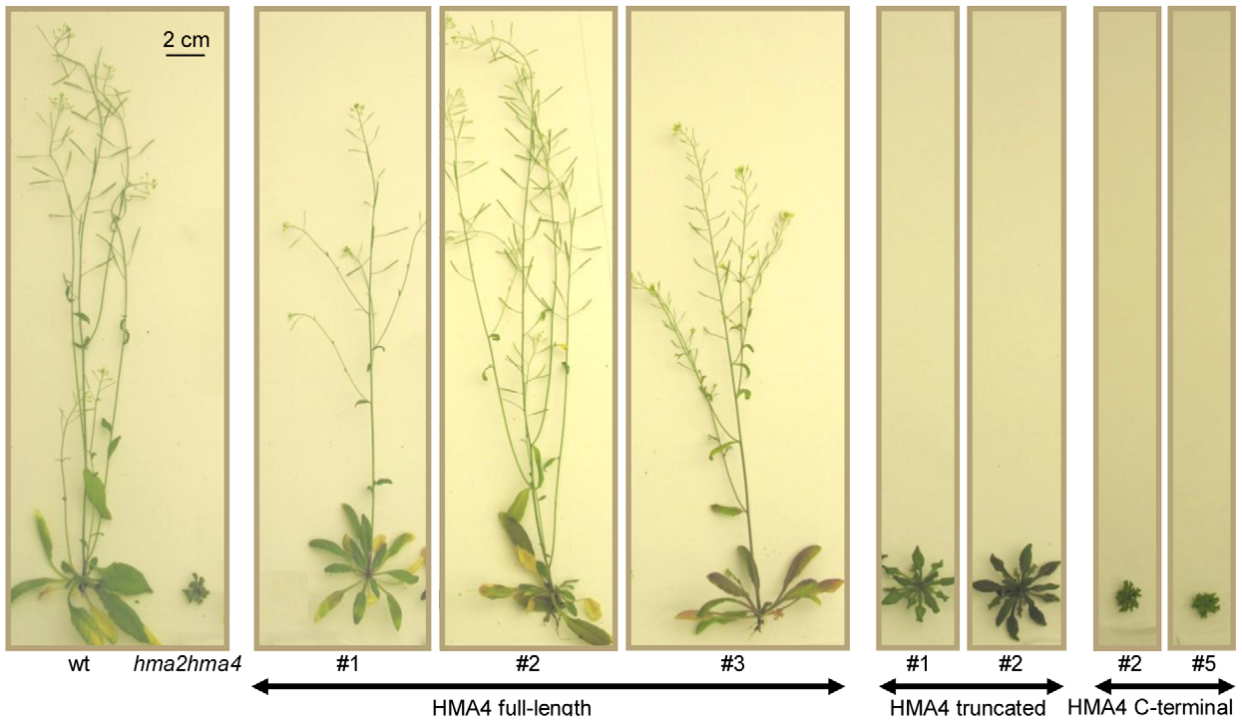
Representative plants of lines expressing *AtHMA4-F*L, *AtHMA4-trunc* or *AtHMA4-C-term*. Plants were grown on soil for 42 days under identical conditions in a controlled-environment growth room (22°C 16 h light, 20°C 8 h dark cycle).

*AtHMA4* expressed from the 35S promoter rescued the stunted growth phenotype of the *hma2 hma4* mutant (Figures 4 and 5). The rosette diameters and bolt heights are shown for 5 independent lines (Figure 4, HMA4-FL). The extent of rescue varied but in all cases transformant rosette sizes and bolt heights were significantly greater than the average value for the *hma2 hma4* mutant. *AtHMA4-trunc* expressed from the 35S promoter gave some rescue of the *hma2 hma4* mutant but the rescue was far less than for lines expressing *AtHMA4-FL* (Figures 4 and 5). In two of the five lines the rosette diameter was significantly greater than the average for the *hma2 hma4* mutant but the *AtHMA4-trunc* transformants did not restore the phenotype fully and few bolts were produced in any of these lines. Expression of *AtHMA4-C-term* did not have any clearly observable effect on growth of the *hma2 hma4* mutant; the rosette diameters were generally similar to the *hma2 hma4* mutant and, similarly, bolts were rarely produced (Figures 4 and 5). Siliques were produced in the *AtHMA4-FL*-expressing lines only and silique and seed measurements were taken in two of the lines. Silique length and also seed number per silique were both slightly but significantly smaller than wt in both AtHMA4-FL lines (Figure S3).

### Effect of expressing *AtHMA4* versions under the 35S promoter on the ionome of the *Arabidopsis hma2 hma4* mutant

**Figure 6:**
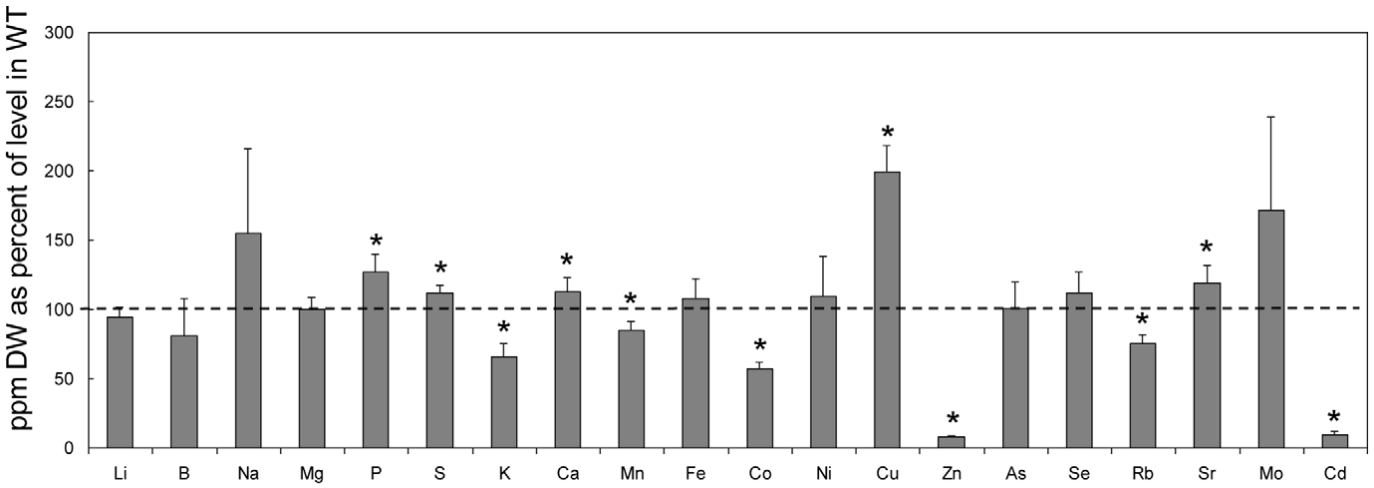
Ionomic profile of *hma2 hma4* mutant. Elemental levels in the shoot of the *hma2 hma4* mutant. Values are the mean +/− S.E of 4 replicate experiments, expressed as % of values for wt (dashed line indicates wt level at 100%). Student’s t-test was used to determine significance levels (P<0.05).

The shoot ion content of the *hma2 hma4* mutant grown on soil was compared with the wt ionome (Figure 6). The results are presented for each element as a percentage of wt values. As reported previously the mutant is markedly deficient in shoot Zn [12] and Cd [14]. However, considering those elements that differ significantly by more or less than 30% of the wt level, there are also other notable differences: Co is markedly reduced; Rb and K are also reduced to a lesser extent, while Cu is markedly higher. There were also smaller but significant differences in a number of other elements. The ionomic profile for the single *hma2* and *hma4* knockout mutants measured in this study and also those available in the Purdue Ionomics database (www.ionomicshub.org) is shown in Figure S4. Consistent with previous reports, Zn is reduced in the shoots of the *hma4* mutants but not to the extent observed in the *hma2 hma4* double mutant. Cd and Co were not always significantly different from wt although they were always lower. Interestingly the three HMA4 TDNA insertion lines that are predicted to contain insertions in the promoter region had ionomic profiles that were comparable with the lines with the TDNA insertion after the start codon (Figure S4A). There was little difference in the ionomic profiles of the *hma2* mutants compared to wt (Figure S4B).

**Figure 7:**
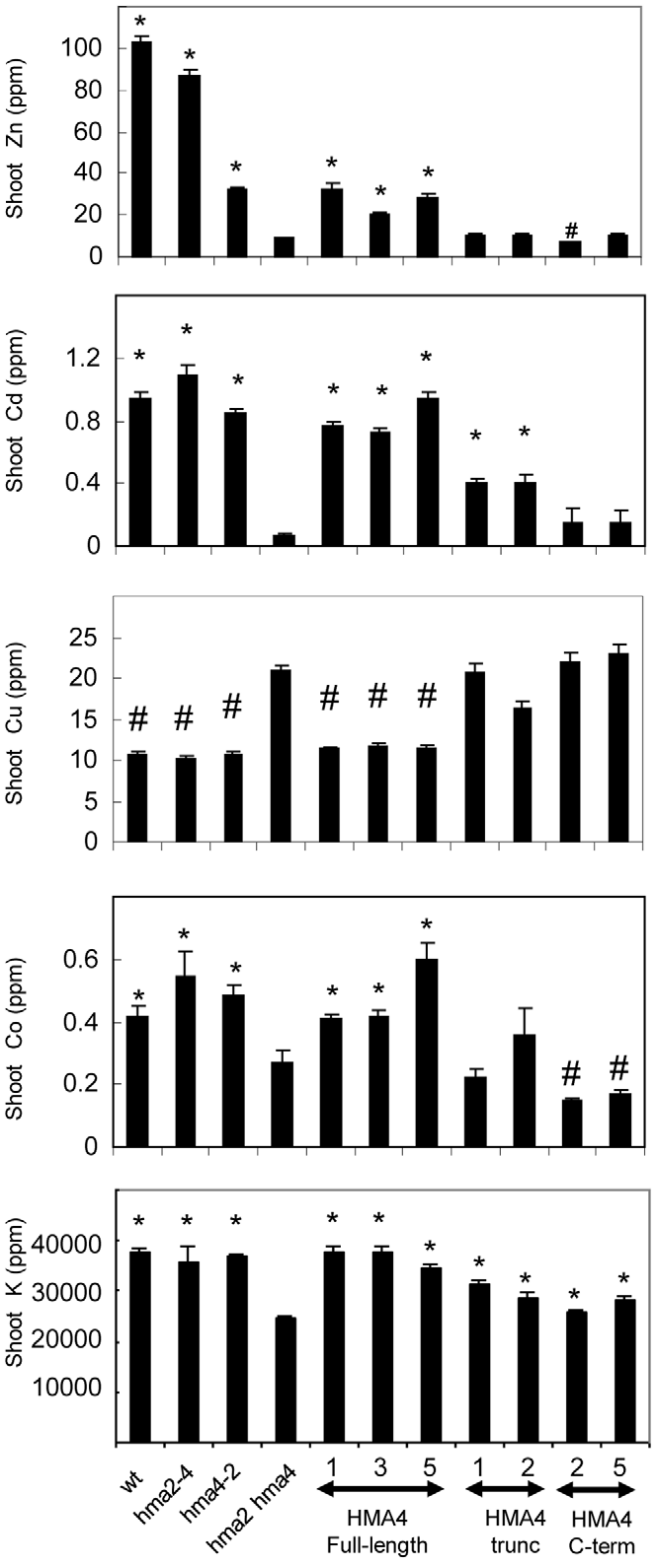
Metal levels in selected lines expressing *AtHMA4-FL*, *AtHMA4-trunc* or *AtHMA4-C-term*. Shoot levels of Zn, Cd, Cu, Co and K are compared in selected lines for *hma2 hma4* plant lines expressing either *AtHMA4-FL*, *AtHMA4-trunc* or *AtHMA4-C-term*. Untransformed *hma2 hma4* controls plants are shown together with wild-type (wt) and *hma2* and *hma4* mutants. Values are the mean +/− S.E. determined from 12 plants. Student’s t-test was used to determine significance levels. * significantly greater than *hma2 hma4* control; # significantly lower than *hma2 hma4* control (P<0.05).

The shoot ion content of several of the lines expressing *AtHMA4* constructs was measured to determine whether the lines showing a rescue of growth also showed a restoration of ion content to wt levels. Results for all elements measured in these lines shown as a percentage of wt are given in Figure S5. For those elements which did show a marked difference when comparing wt and the *hma2 hma4* mutant, the levels measured are shown in Figure 7. The results are displayed for the *hma2 hma4* mutant, *hma2* and *hma4* single mutants and several lines expressing *AtHMA4 FL* or *AtHMA4-trunc* which had shown a rescue to some extent of the growth phenotype. Two lines expressing *AtHMA4-C-term* were also included in this analysis for comparison. *AtHMA4-FL* expressed from the 35S promoter showed a partial restoration of the Zn content to approximately 30% of wt levels; Cd contents were restored to approximately 80% of wt levels. Co was fully restored to wt levels, and Cu was reduced to wt levels. For the *AtHMA4-trunc* transformants, Cd levels were significantly elevated compared to the *hma2 hma4* background mutant, but Zn, Co and Cu levels did not differ significantly from *hma2 hma4* levels (Figure 7). Cd and Cu levels did not differ significantly between the *AtHMA4-C-term* transformants and the background mutant; however Co levels were significantly lower in the *AtHMA4-C-term* transformants (Figure 7). For one of the two *AtHMA4-C-term* transformants, Zn levels were no different from the background mutant, but in the other they were slightly but significantly lower. In both of the *AtHMA4-C-term* transformant lines, Cd levels were higher than in the background mutant, but the differences were not significant (Figure 7).

## Discussion

In devising strategies for improving the Zn content of crops we may consider ectopically expressing transporters known to be involved in transferring Zn to the shoot. It may also be possible to modify these transporters in order to achieve more efficient transfer. The importance of AtHMA4 in transport of Zn to the shoot has previously been shown in *Arabidopsis* [12], and highly homologous proteins are central to Zn accumulation in the hyperaccumulators *A. halleri* [18] and *Thlaspi caerulescens* [16], [21]. This study has investigated AtHMA4 in more detail and tested different versions of this pump both in yeast and in plants to learn more about their potential in biofortification or phytoremediation strategies.

### AtHMA4-trunc and AtHMA4 C-term are more effective than AtHMA4 FL in conferring Zn and Cd tolerance to yeast

Heterologous expression in yeast with growth analysis at pH 5–5.5 shows that the full length *AtHMA4*, the truncated variant, or the cytoplasmic C-terminus alone all result in Zn and Cd tolerance, although the levels of yeast growth differ. For Cd, the relative levels of tolerance conferred by these *AtHMA4* constructs to wt yeast was *AtHMA4-C*-term > *AtHMA4-trunc* > AtHMA4-FL. In yeast, the vacuolar ABC transporter YCF1 transports glutathione-conjugated Cd for detoxification [22]. However all three *AtHMA4* constructs enhanced Cd tolerance in the *ycf1* mutant, indicating that YCF1 protein is not required for detoxification mediated by these *AtHMA4* variants. There was a slight difference in the order of Zn tolerance conferred on Zn-sensitive *zrc1 cot1* yeast: AtHMA4-trunc > AtHMA4-C-term > AtHMA4-FL. The truncated form of the pump retains the transmembrane domains that catalyse transmembrane metal transport and so is also likely to catalyse efflux in the same manner as AtHMA4-FL, and confer tolerance to metals in this way. The fact that it is more effective than the full-length version suggests that deletion of the C-terminus results in a more efficient pump in conferring Zn and Cd tolerance to yeast. This could be because removal of the C-terminus increases the transport rate of AtHMA4, or it could result in a different localisation for the AtHMA4-trunc protein compared to the AtHMA4-FL, which could be sufficient to alter the tolerance conferred without any change in transport rate. Recently it was shown that deletion of the C-terminus from AtHMA4 had no apparent effect on enzyme turnover but this resulted in more efficient Zn or Cd pumping; it was suggested that slipping may account for this as has been observed for certain other P-type ATPases [23].The AtHMA4-C-terminal region does not include any catalytic or predicted transmembrane domains, so we postulate that when expressed alone it may act as a metal-binding peptide to mediate Cd and Zn detoxification conferring metal tolerance. Consistent with this, the C-terminal region has been shown *in vitro* to bind both Zn and Cd [23]. The 473 amino acid C-terminal region has 45 Cys residues, including 13 CC motifs. Cys residues feature in Cd tolerance proteins such a phytochelatins [24], metallothioneins [25] and AtPCR proteins [26]. Therefore when expressed in unicellular organisms AtHMA4-C-term could have potential biotechnological application in bioremediation strategies.

### The ionome of the *hma2 hma4* mutant is markedly different to wt plants

Ionomics helps us understand the relationship between different elements and the responses of the plant to environmental conditions at various stages of growth and development [27]. It is also useful in understanding effects of genetic modification. Our goal was to determine whether the truncated version of AtHMA4 was also more efficient *in planta*; in particular, whether it could be more effective than the FL version in allowing Zn accumulation in the shoot. We used the *hma2 hma4* mutant which is defective in Zn and Cd root to shoot translocation [12], [14] in order to analyse the AtHMA4 variants. In particular we were interested in how these mutants and transgenic lines would perform in the more natural soil environment, rather than growing on plates or in hydroponics. The *hma2 hma4* double knockout mutant accumulates Zn in the pericycle and endodermal cells of the root, and the mutant is stunted unless grown with additional Zn supplementation [12], [28]. Our analysis of the *hma2 hma4* shoot ionome confirms that Zn and Cd levels are extremely low compared to wt, and additionally shows there are lower levels of Co, K and Rb. The *hma2 hma4 mutant* also shows significantly higher Cu levels in the shoots. Shoots of the single *hma4* knockouts are also slightly lower in Co, although their K and Cu contents are normal. There is substantial evidence that HMA4 transports Zn and Cd, so it is not unexpected that these metals are decreased in the *hma2 hma4* double mutant. HMA4 may also transport Co [13], [20], and its absence could result in the decrease in Co observed in both the single *hma4* mutant and the double knockout. There is no evidence that AtHMA4 can transport Cu but under Zn deficiency many other transporters (eg ZIPs 2, 4 5 and 9 and also COPT2) are up-regulated, and some of these can transport Cu as well as Zn [29]. So the observed increase in shoot Cu may be due to a Zn deficiency response whereby such Zn transporters are up-regulated, resulting in Cu uptake. It is interesting that elevated Cu is only seen in the *hma2 hma4* mutant, which has Zn levels around 10% of that observed in wt plants, but not in the single *hma4* mutants that have Zn levels around 40% of wt plants. This suggests that there could be a threshold level of Zn below which transporters are induced that lead to the accumulation of Cu in the shoots. K and Rb are also decreased in the *hma2 hma4* mutant. There is no evidence for K transport by HMA2 or HMA4, so the decreased K levels observed in the *hma2 hma4* knockouts are more likely to be an indirect result of the absence of these transporters, although we do not know what this pathway involves. Rb has no known biological function but has a similar ionic radius to K and can be used as a K analogue. It seems likely that the observed decrease in Rb is directly related to the decrease in K.

### Significant restoration of the wt phenotype in *hma2 hma4* plants expressing 35Sp-*AtHMA4*

In most of the lines with *AtHMA4-FL* expressed under the 35S promoter the small rosette phenotype of *hma2 hma4* double knockout plants was fully restored to the size observed for wt plants or the *hma2* and *hma4* single mutants. Flowering bolts were also produced in the *AtHMA4-FL* transformants and all lines had a significantly greater mean bolt height than the *hma2 hma4* mutant. In three of the five lines the mean height was similar to that observed for the *hma2* single mutant, but it was still significantly less than wt plants indicating that it was not a complete rescue in all plants. Siliques and seed were also produced in the *AtHMA4-FL*-expressing lines but both silique length and also seed number per siligue were slightly but significantly smaller than wt (Figure S3). It should be noted that expression of *AtHMA4-FL* only restored the shoot Zn content to around 30% of the level observed in wt plants. This Zn content is similar to the levels seen in the *hma4* mutant, which appears similar to wt in most growth parameters. Thus less than half the normal shoot Zn content is required for typical growth. In contrast Co and Cu levels were restored to wild-type levels in these lines. The smaller silique size and lower seed number per silique of the *AtHMA4 FL*-transformants compared to wild-type is likely to be a consequence of the reduced Zn levels in the shoot and hence lower levels reaching the silique. However it could be that HMA4 plays a more direct role in the siliques themselves and expression under the 35S promoter is not as effective here as under the native promoter.

The reason why Zn content is only restored to levels seen in the *hma4* mutant and not to wt levels is not known. It is possibly due to mis-expression of HMA4 due to its expression under the 35S promoter. This may result in less efficient translocation of Zn to the shoot. It would be interesting in the future to see if similar responses in growth and Zn content are seen in hydroponically grown plants and in that case root measurements could be included to determine whether root accumulation differs in transformants and wt. Certainly when *AtHMA4-FL* was expressed in wt Arabidopsis (Col ecotype) there was no change in root Zn but increased shoot Zn under hydroponic growth conditions, indicating increased root to shoot translocation (13). However when expressed in tobacco, Zn concentrations were either unaltered in roots (0.5, 10 µM Zn supply) or reduced (100, 200 µM Zn supply) while maintaining or increasing (only at 10 µM Zn supply) shoot content, indicating that root to shoot translocation of Zn was greater in tobacco expressing *35S-AtHMA4-FL* than in wt (17).

### Functional significance of C-terminal region of AtHMA4

The C-terminus of AtHMA4 would not be predicted to function as a metal transporter and so when expressed in *hma2 hma4* we would predict that it would not be directly involved in translocation of Zn or Cd to or from the shoot. However as it confers metal tolerance on yeast it may function as a metal-binding peptide, and could influence metal levels when expressed in plants. We tested whether expression of *AtHMA4-C-term* had any effect on the ionomic profile. No marked effect was observed in most elements although in one line the Zn content was slightly lower and in both lines the Co concentration was slightly lower. There was also a trend towards increased Cd in these lines although this was not significant. Generally no significant difference in the growth of the C-terminal-expressing *hma2 hma4* lines compared to the *hma2 hma4* mutant was observed. It should be noted that expression of the C-terminus of AtHMA4 in wt tobacco exposed to 0.5 or 5 µM Zn or 0.25 µM Cd resulted in Zn or Cd accumulation in roots and shoots [17] indicating that this may have future biotechnological application.

In contrast to the results obtained for *AtHMA4-FL*, *AtHMA4-trunc* expressed under the 35S promoter was not very effective in restoring the phenotype of the *hma2 hma4* mutants and in restoring the shoot Zn content of this mutant. Similarly Co and Cu levels were no different from the *hma2 hma4* mutant, but *AtHMA4-trunc* expression did seem to increase shoot Cd content, although this was still significantly lower than wild-type levels. These results suggest that in terms of developing strategies for Zn biofortification, the truncated version of AtHMA4 would not be as effective as the FL version. This is consistent with results obtained expressing *AtHMA4-trunc* in tobacco [17]. The possibility exists that AtHMA4-trunc is more effective than AtHMA4 FL in the roots and could function in metal exclusion when expressed under the 35S-promoter. This may explain the lower accumulation of Zn and Cd in the shoot. Alternatively it could influence root sequestration of these metals with less transfer to shoots. In future it would be interesting to determine the effect on the root ionome in mutants expressing these constructs and also determine the cellular localisation pattern.

Our results suggest that the C-terminus of AtHMA4 does have an important role *in planta* and its removal from AtHMA4 FL does have consequences for the functioning of AtHMA4. This is in contrast to what has been observed for AtHMA2 where C-terminally truncating this pump had little effect; the truncated version of this pump rescued the Zn deficiency stunted phenotype of the *hma2 hma4* mutant as well as the full-length version [15]. The sterility phenotype was however not rescued in the AtHMA2 version with the entire C-terminus deleted. The reason for the differences observed in the ability of C-terminally truncated versions of AtHMA2 and AtHMA4 in rescuing the *hma2 hma4* mutant is not clear. *AtHMA2* and its C-terminally-truncated version were expressed under their native promoter whereas in this study all constructs were expressed under the 35S promoter and we cannot exclude this as a possible reason for the difference. The C-termini of AtHMA2 and AtHMA4 do vary in size and composition with AtHMA2 having a much shorter C-terminal domain (260aa compared with 470aa after the last predicted transmembrane domain). Cys and His residues have been identified as metal-binding residues in the C-terminus of AtHMA2, binding three Zn^2+^ ions with high affinity (*K*d ∼16 nM). Three His and a Cys coordinate the one Zn ion, while His residues alone co-ordinate the other 2 ions [19]. Although deleting the C-terminal region of AtHMA2 had little effect on its ability to restore the Zn-deficiency phenotype of *hma2 hma4*, deletion of its C-terminal region has been shown to half the activity of the pump without significantly altering Zn^2+^ or Cd^2+^ *K*_1/2_ for ATPase activation [19]. This was interpreted as an auto stimulatory mechanism for AtHMA2 whereby cytoplasmic metal binding to the C-terminus drives faster transport [19]. The increased Zn and Cd tolerance in yeast conferred by removal of the C-terminal region of AtHMA4 is more consistent with an auto inhibitory role for the C-terminus in this pump although further analysis is required to investigate this. Certainly the C-terminus of AtHMA4 does seem to have an important role *in planta* but there is no evidence as yet for an auto inhibitory function. In fact considering accumulation of Zn in the shoots, removal of the C-terminus seems to result in a less effective pump. There are several possible reasons for AtHMA4-trunc being less effective than AtHMA4-FL in rescuing the Zn-deficiency phenotype: the truncated version of the protein could be less stable when expressed in plants; it may be targeted to a different membrane; it could function more efficiently as a metal efflux system in roots so that metals are transferred out of the plant. Although important *in planta*, the role of the C-terminus is still not clear; it could act as a metal sensor to regulate activity of the pump in response to available ions or it may interact with proteins that regulate the pump, or with a metal chaperone to specifically target Zn to the pump for transport (although no Zn chaperone has yet been identified). Any differences in the operation of these constructs in yeast and plants could be due to several factors: targeting signals may differ, turnover rates may vary, and interactions with other proteins and with the endogenous metal-responsive transcriptome may differ between yeast and plants.

In conclusion, it seems that AtHMA4 FL is more promising for future biotechnological application than AtHMA4-trunc and that complementation of the *hma2 hma4* mutant is a suitable strategy for exploring structure/function relationships of AtHMA4.

## Materials and Methods

### Plant material and growth conditions

*Arabidopsis thaliana* ‘Columbia-8’ (European Arabidopsis Stock Centre N60000; http://arabidopsis.info/) and transformed lines were grown in a controlled-environment growth room (22°C 16 h light, 20°C 8 h dark cycle) or under similar conditions in a glasshouse, in 1∶1∶1 (v/v) JI No. 2: Vermiculite (Medium): VAPOGRO SEED MODULAR (Winscombe, UK), with 0.28 g/L INTERCEPT 5 g insecticide (Bayer, Canada). *Arabidopsis thaliana* (Columbia) *hma2-4* (SALK_034393) *hma4-2* (SALK_050924) double T-DNA insertion mutant [14], [15] was a kind gift from Prof. C. Cobbett. The *hma2 hma4* mutant plants are infertile under normal growth conditions, therefore Zn supplementation was provided by watering with 3 mM ZnCl_2_ in order to grow *hma2 hma4* plants for transformation. This was also supplied to isolate seed from transformed *hma2 hma4* but not in the phenotypic analysis unless stated specifically.

Growth parameters (rosette diameter and bolt height) were determined after 42 days growth. For silique measurements, photographs of siliques were taken after 49 days growth of plants on soil and representative bolts were taken from each line. Silique lengths were determined from these pictures using ImageJ software (http://rsbweb.nih.gov/ij/). Silique lengths were measured for 60 siliques from six plants in the middle section of the bolt. To measure seed per silique, the siliques were immersed in 70% ethanol (v/v) overnight and then transferred into methyl salicylate (100%) and left overnight. This produced clear siliques which were photographed under the microscope allowing seeds to be counted.

### *AtHMA4* constructs for yeast expression, yeast transformation and growth analyses

The full length *AtHMA4* coding sequence (AtHMA4-FL, 1172 aa), and the truncated *AtHMA4* sequence lacking the C-terminal region (Δ714-1172, AtHMA4-trunc, 713 aa) were cloned into the yeast expression vector p426 under control of a galactose inducible promoter as described previously [10], [11]. The C-terminal 473 amino acids of *AtHMA4* were amplified using primers 5′GAACTAGTAGGGACTTGTCTGCTTGTGA and 5′GTATCGATGGCATTCACGGAATGAGACT, digested with SpeI and ClaI, and inserted into same sites of p426. This allows expression of the deletion mutant Athma4Δ1–699, referred to as AtHMA4-C-term. Constructs were transformed into wt *Saccharomyces cerevisiae* (BY4741), the *ycf1* mutant, or the *zrc1 cot1* double mutant as described previously [11]. For metal sensitivity tests yeast cells were grown in liquid culture overnight at 30°C in SC (Synthetic Complete) without uracil (5 g L^−1^ (NH_4_)_2_SO_4_, 1.7 g L^−1^ yeast nitrogen base (Difco, UK), 1.92 g L^−1^ yeast synthetic drop-out media supplement without uracil; (Sigma, UK)) and containing 2% (w/v) glucose (adjusted to pH 5.0 with KOH prior to autoclaving). The cultures were diluted to an OD_600_ of approximately 0.8 with SC without uracil containing 2% (w/v) galactose (pH 5.0) and grown for a further 4 h. The cultures were diluted to the same OD_600_ (approx. 0.3) and aliquots were inoculated onto SC without uracil, 2% (w/v) agar (Difco technical) 2% (w/v) galactose (adjusted to pH 5.0 with KOH before addition of agar and prior to autoclaving) containing various concentrations of metal supplied as the sulphate salt. All culture dilutions were made in SC without uracil, 2% (w/v) galactose (pH 5.0). Final pH measurements were made after autoclaving. Inoculated plates were incubated at 30°C for 3–5 days.

### Generation of *AtHMA4* constructs for expression in plants

Full-length *AtHMA4*, the truncated *AtHMA4* mutant or the 473 amino acid *AtHMA4* C-terminal region were inserted into the expression vector pBECKS400.6 [30] under control of the CaMV35S promoter. *AtHMA4-FL* was EcoRI-digested from p426, the ends were filled and the sequence was ligated into the SmaI site of pBECKS400.6. *AtHMA4-trunc* was amplified with primers 5′CTCGGATCCGAAAATGGCGTTACAAAACAAAG and 5′GCGGTACCTCACTTTTTGTTCCCAATCTTTTTCTTCTCTC, digested with BamHI and KpnI and inserted into the same sites of pBECKS400.6. The reverse primer introduces a stop codon. *AtHMA4-C-term* was amplified with primers 5′CTAGTAGGGACTTGTCTGCTTGTGA and 5′CGATGGCATTCACGGAATGAGACT, and ligated into the SmaI site of pBECKS400.6. The ATG at bp 2098 of the *AtHMA4* coding sequence starts expression of the C-terminal 473 amino acids of AtHMA4.

### Plant transformation

Plasmids were transformed into *Agrobacterium tumefaciens* GV3101 by electroporation. *Arabidopsis thaliana* (Columbia) *hma2-4 hma4-2* mutant [12] was grown with Zn supplementation to promote flowering, and plants were transformed using the floral dip method but including a 3 h pre-induction of *vir* genes by addition of 100 µM acetosyringone to the culture before dipping [31]. Homozygous T3 plants were used for analysis.

### RT-PCR

RNA and cDNA were prepared and semi-quantitative PCR was performed as described by [32]. Actin 2, used as the control, was amplified using primers 5′GGTAACATTGTGCTCAGTGGTGG and 5′CTCGGCCTTGGAGATCCACATC that span an intron. Two alternative primer pairs were used to detect *AtHMA4-FL*. Primers 5′GGAATTCGCAGCAGTTGTGTTCCTATTCA and 5′GGAATTCGAGATTTGGTTTTACTGCTCTG detect a region before the C-terminus and also amplify *AtHMA4-trunc* but do not amplify *AtHMA4-C-term*, while primers 5′GAAGGAGCAATGTCGTCTGGAG and 5′AGCACTCACATGGTGATGGT detect a region within the C-terminus and so also amplify *AtHMA4-C-term* but not *AtHMA4-trunc*.

### Ionomic analysis

Plants were grown in soil supplemented with sub-toxic concentrations of various elements including 0.09 ppm Cd and were regularly watered with Fe-HBED and 0.25× Hoagland’s solution [33]. Elemental analysis was carried out as previously described using ICP-MS [33]. Ionomic data is available at www.ionomicshub.org; tray references for the single mutants are 260 (Ws hma2-1), 816 (SALK_042906, SALK_093482, SALK_109431 and SALK_034393), 940 (GABI_168C10), 1609 (SALK_050924) and 1615 (SALK_132258, SALK_066029 and SALK_019060).

## Supporting Information

Figure S1 **Schematic diagram of AtHMA4-FL protein and equivalent schematic diagrams of the two partial sequences AtHMA4-trunc and AtHMA4-C-term.** Predicted transmembrane domains are shown as cylinders. The location corresponding to the HMA4-2 TDNA insertion site is indicated, as are the sites for the two point mutations D401A and C357G.
(0.44 MB TIF)

Figure S2 **Alignment of HMA4 with HMA2 and HMA3, showing AtHMA4-trunc and AtHMA4-C-term ORFs, primer locations and HMA4-2 TDNA insertion.** ClustalW2 (UPGMA clustering) alignment of AtHMA4 (At2g19110) with AtHMA2 (At4g30110) and AtHMA3 (At4g30120) cDNA sequences. Conserved residues are shaded. The ORFs (with stop codons) for 35S expression constructs AtHMA4-trunc (AtHMA4trun) and AtHMA4-C-term (AtHMA4Cter) are shown below the alignment in green and yellow respectively. Primers that amplify a region of the sequence before the C-terminus are indicated in dark blue; primers that amplify a region of the sequence within the C-terminus are indicated in light blue. The HMA4-2 TDNA insertion position maps to the cDNA just after C600, indicated in red.
(0.14 MB TIF)

Figure S3 **Silique lengths and number of seeds per silique is reduced in the AtHMA4-FL lines.** Silique lengths (A) and number of seeds per silique (B) taken from lines after 49 days growth on soil. Plants were watered with tap water apart from the +Zn plants which were watered with 3 mM ZnSO4. Silique lengths are the mean ± S.E. of 60 siliques from six plants while values for seed per silique were determined from 36–40 siliques from five plants. Significant differences are indicated: # significantly greater than wt; * significantly lower than wt (Students t test, P<0.05). Example siliques are shown for wt (C), *hma2 hma4* + Zn (D), AtHMA4-FL line 2 and AtHMA4 FL line 3. Scale bar  = 1 mm.
(0.86 MB TIF)

Figure S4 **Ionomic profiles for shoots of T-DNA insertion lines.** A, *hma2* mutants; B, *hma4* mutants. Values are the mean +/− S.E. (n = 12) expressed as % of values for wt.
(0.66 MB TIF)

Figure S5 **Ionomic profiles for shoots of selected lines of hma2 hma4 expressing either AtHMA4-FL, AtHMA4-trunc or AtHMA4-C-term.** Values are the mean +/− S.E. determined from 12 plants expressed as % of values for wt.
(0.47 MB TIF)

## References

[1] Copenhagen Consensus 2008 website (accessed 2010). http://www.copenhagenconsensus.com/Home.aspx

[2] White PJ, Broadley MR (2009). Biofortification of crops with seven mineral elements often lacking in human diets - iron, zinc, copper, calcium, magnesium, selenium and iodine.. New Phytol.

[3] Brown KH, Peerson JM, Allen LH (1998). Effect of zinc supplementation on children's growth: A meta-analysis of intervention trials.. Bibl Nutr Dieta.

[4] Caulfield LE, de Onis M, Blossner M, Black RE (2004). Undernutrition as an underlying cause of child deaths associated with diarrhoea pneumonia malaria and measles.. Am J Clin Nutrition.

[5] Brown KH, Peerson JM, Baker SK, Hess SY (2009). Preventive zinc supplementation among infants preschoolers and older prepubertal children.. http://www.zinc-health.org/G_H_Docs/Health_Documents/food_nutrition_bulletin_2009.pdf.

[6] Bhutta ZA, Black RE, Brown KH, Gardner JM, Gore S (1999). Prevention of diarrhoea and pneumonia by zinc supplementation in children in developing countries: Pooled analysis of randomized controlled trials.. JPediatr.

[7] Palmgren MG, Clemens S, Williams LE, Krämer U, Borg S (2008). Zinc biofortification of cereals; problems and solutions.. Trends Plant Sci.

[8] Ramesh SA, Choimes S, Schachtman DP (2004). Over-expression of an Arabidopsis zinc transporter in *Hordeum vulgare* increases short-term zinc uptake after zinc deprivation and seed zinc content.. Plant Mol Biol.

[9] Genc Y, Verbyla AP, Torun AA, Cakmak I, Willsmore K (2009). Quantitative trait loci analysis of zinc efficiency and grain zinc concentration in wheat using whole genome average interval mapping.. Plant Soil.

[10] Mills RF, Krijger GC, Baccarini PJ, Hall JL, Williams LE (2003). Functional expression of AtHMA4 a P_1B_ATPase of the Zn/Co/Cd/Pb subclass.. Plant J.

[11] Mills RF, Francini A, daRocha PSCF, Bacarini PJ, Aylett M (2005). The plant P-1B-type ATPase AtHMA4 transports Zn and Cd and plays a role in detoxification of transition metals supplied at elevated levels.. FEBS Lett.

[12] Hussain D, Haydon MJ, Wang Y, Wong E, Sherson SM (2004). P-type ATPase heavy metal transporters with roles in essential zinc homeostasis in Arabidopsis.. Plant Cell.

[13] Verret F, Gravot A, Auroy P, Leonhardt N, David P (2004). Overexpression of AtHMA4 enhances root-to-shoot translocation of zinc and cadmium and plant metal tolerance.. FEBS Lett.

[14] Wong CK, Cobbett CS (2009). HMA P-type ATPases are the major mechanism for root-to-shoot Cd translocation in *Arabidopsis thaliana*.. New Phytol.

[15] Wong CK, Jarvis RS, Sherson SM, Cobbett CS (2009). Functional analysis of the heavy metal binding domains of the Zn/Cd-transporting ATPase HMA2 in *Arabidopsis thaliana*. New Phytol.

[16] Bernard C, Roosens N, Czernic P, Lebrun M, Verbruggen N (2004). A novel CPx-ATPase from the cadmium hyperaccumulator *Thlaspi caerulescens*.. FEBS Lett.

[17] Siemianowski O, Mills RF, Williams LE, Antosiewicz DM (2010). Expression of the P1B-type ATPase AtHMA4 in tobacco modifies Zn and Cd root to shoot partitioning and metal tolerance.. Plant Biotechnol J: In press.

[18] Hanikenne M, Talke IN, Haydon MJ, Lanz C, Nolte A (2008). Evolution of metal hyperaccumulation required cis-regulatory changes and triplication of HMA4.. Nature.

[19] Eren E, Kennedy DC, Maroney MJ, Arguello JM (2006). A novel regulatory metal binding domain is present in the C terminus of Arabidopsis Zn^2+^-ATPase HMA2.. J Biol Chem.

[20] Verret F, Gravot A, Auroy P, Preveral S, Forestier C (2005). Heavy metal transport by AtHMA4 involves the N-terminal degenerated metal binding domain and the C-terminal His stretch.. FEBS Lett.

[21] Papoyan A, Kochian LV (2004). Identification of *Thlaspi caerulescens* genes that may be involved in heavy metal hyperaccumulation and tolerance. Characterization of a novel heavy metal transporting ATPase.. Plant Physiol.

[22] Li ZS, Szczypka M, Lu YP, Thiele DJ, Rea PA (1996). The yeast cadmium factor protein (YCF1) is a vacuolar glutathione S-conjugate pump.. J Biol Chem.

[23] Bækgaard L, Mikkelsen MD, Sørensen DM, Hegelund JN, Persson DP A combined Zn/Cd sensor and Zn/Cd transport regulator in a heavy metal pump.. J Biol Chem. In press.

[24] Clemens S (2006). Evolution and function of phytochelatin synthases.. J Plant Phys.

[25] Robinson NJ, Tommey AM, Kuske CC, Jackson PJ (1993). Plant metallothioneins.. Biochem J.

[26] Song WY, Martinoia E, Lee J, Kim D, Kim D-Y (2004). A novel family of cys-rich membrane proteins mediates cadmium resistance in Arabidopsis.. Plant Phys.

[27] Williams LE, Salt DE (2009). The plant ionome coming into focus.. Current Opinion in Plant Biology.

[28] Sinclair SA, Sherson SM, Jarvis R, Camakaris J, Cobbett CS (2007). The use of the zinc-fluorophore Zinpyr-1 in the study of zinc homeostasis in Arabidopsis roots.. New Phytol.

[29] Wintz H, Fox T, Wu YY, Feng V, Chen W (2003). Expression profiles of *Arabidopsis thaliana* in mineral deficiencies reveal novel transporters involved in metal homeostasis.. J Biol Chem.

[30] McCormac AC, Elliot MC, Chen DF (1997). pBECKS: a flexible series of binary vectors for Agrobacterium-mediated plant transformation.. Mol Biotechnol.

[31] Clough SJ, Bent AF (1998). Floral dip: a simplified method for *Agrobacterium*-mediated transformation of *Arabidopsis thaliana*.. Plant J.

[32] Mills RF, Doherty ML, Lopez-Marques RL, Weimar T, Dupree P (2008). ECA3, a Golgi-localized P-2A-type ATPase plays a crucial role in manganese nutrition in Arabidopsis.. Plant J.

[33] Lahner B, Gong J, Mahmoudian M, Smith EL, Abid KB (2003). Genomic scale profiling of nutrient and trace elements in *Arabidopsis thaliana*.. Nat Biotechnol.

