# Supplementary figures and images for: Functional Significance of AtHMA4 C-Terminal Domain In Planta
Source: PLoS One. 2010 Oct 20;5(10):e13388. doi: 10.1371/journal.pone.0013388 (PMC2958113; doi:10.1371/journal.pone.0013388)

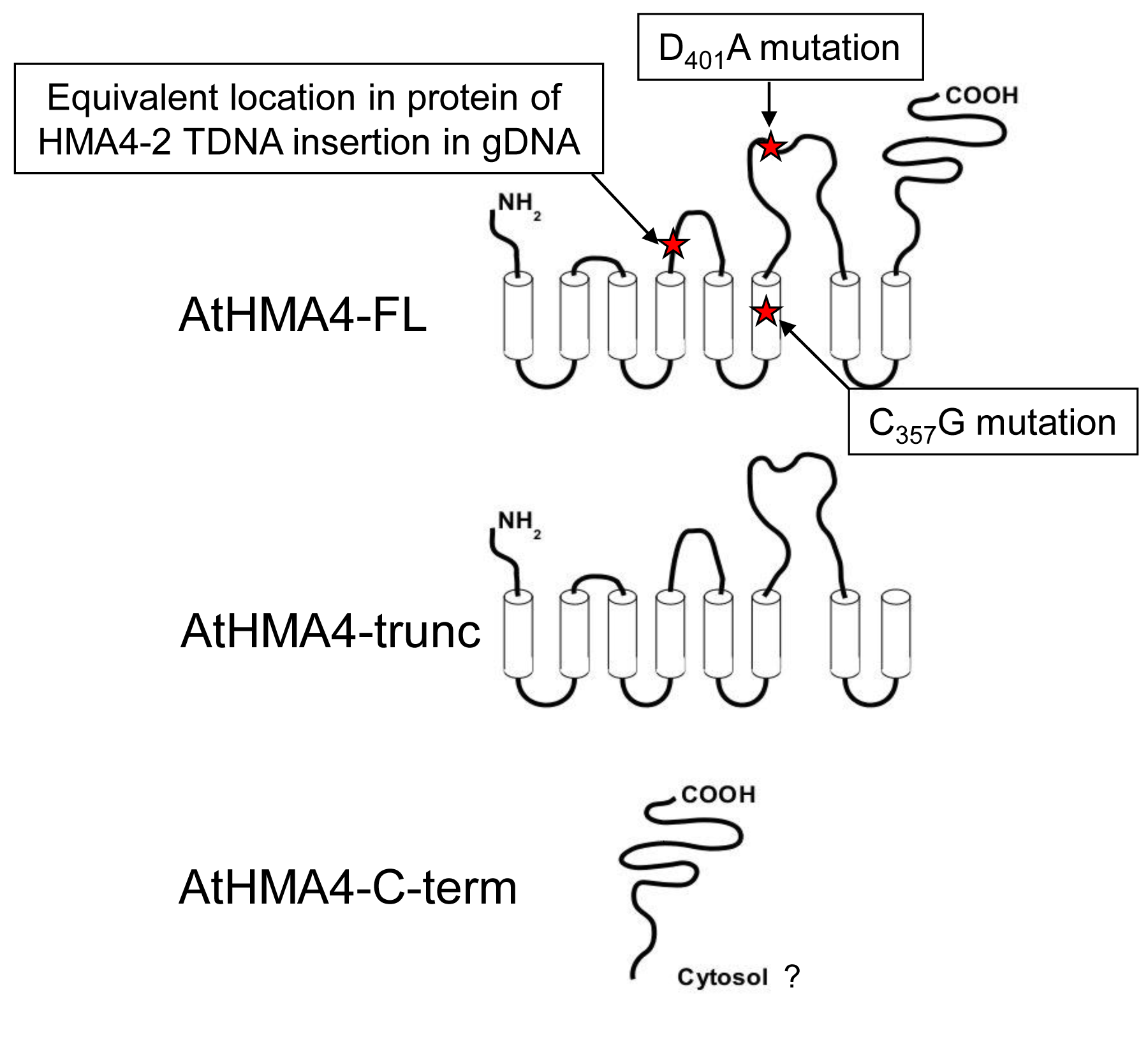

Supplement: Figure S1 — Schematic diagram of AtHMA4-FL protein and equivalent schematic diagrams of the two partial sequences AtHMA4-trunc and AtHMA4-C-term. Predicted transmembrane domains are shown as cylinders. The location corresponding to the HMA4-2 TDNA insertion site is indicated, as are the sites for the two point mutations D401A and C357G. (0.44 MB TIF) [file pone.0013388.s001.tif]

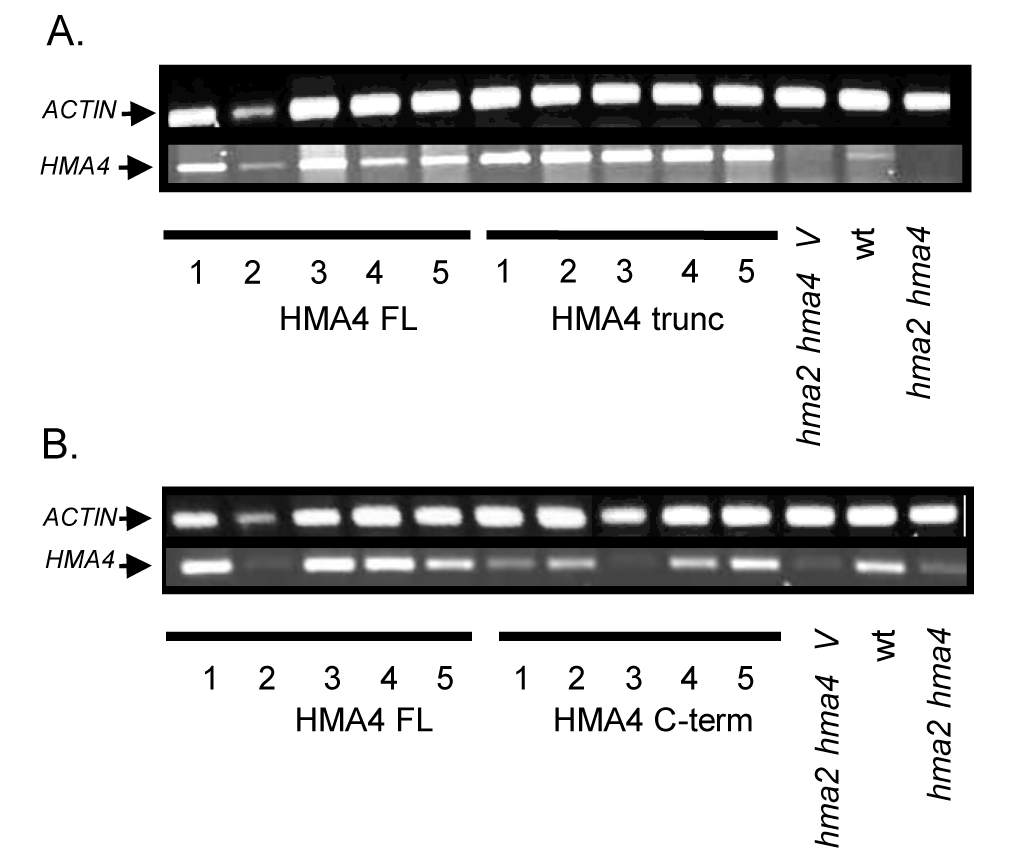

Supplement: Figure S2 — Alignment of HMA4 with HMA2 and HMA3, showing AtHMA4-trunc and AtHMA4-C-term ORFs, primer locations and HMA4-2 TDNA insertion. ClustalW2 (UPGMA clustering) alignment of AtHMA4 (At2g19110) with AtHMA2 (At4g30110) and AtHMA3 (At4g30120) cDNA sequences. Conserved residues are shaded. The ORFs (with stop codons) for 35S expression constructs AtHMA4-trunc (AtHMA4trun) and AtHMA4-C-term (AtHMA4Cter) are shown below the alignment in green and yellow respectively. Primers that amplify a region of the sequence before the C-terminus are indicated in dark blue; primers that amplify a region of the sequence within the C-terminus are indicated in light blue. The HMA4-2 TDNA insertion position maps to the cDNA just after C600, indicated in red. (0.14 MB TIF) [file pone.0013388.s002.tif]

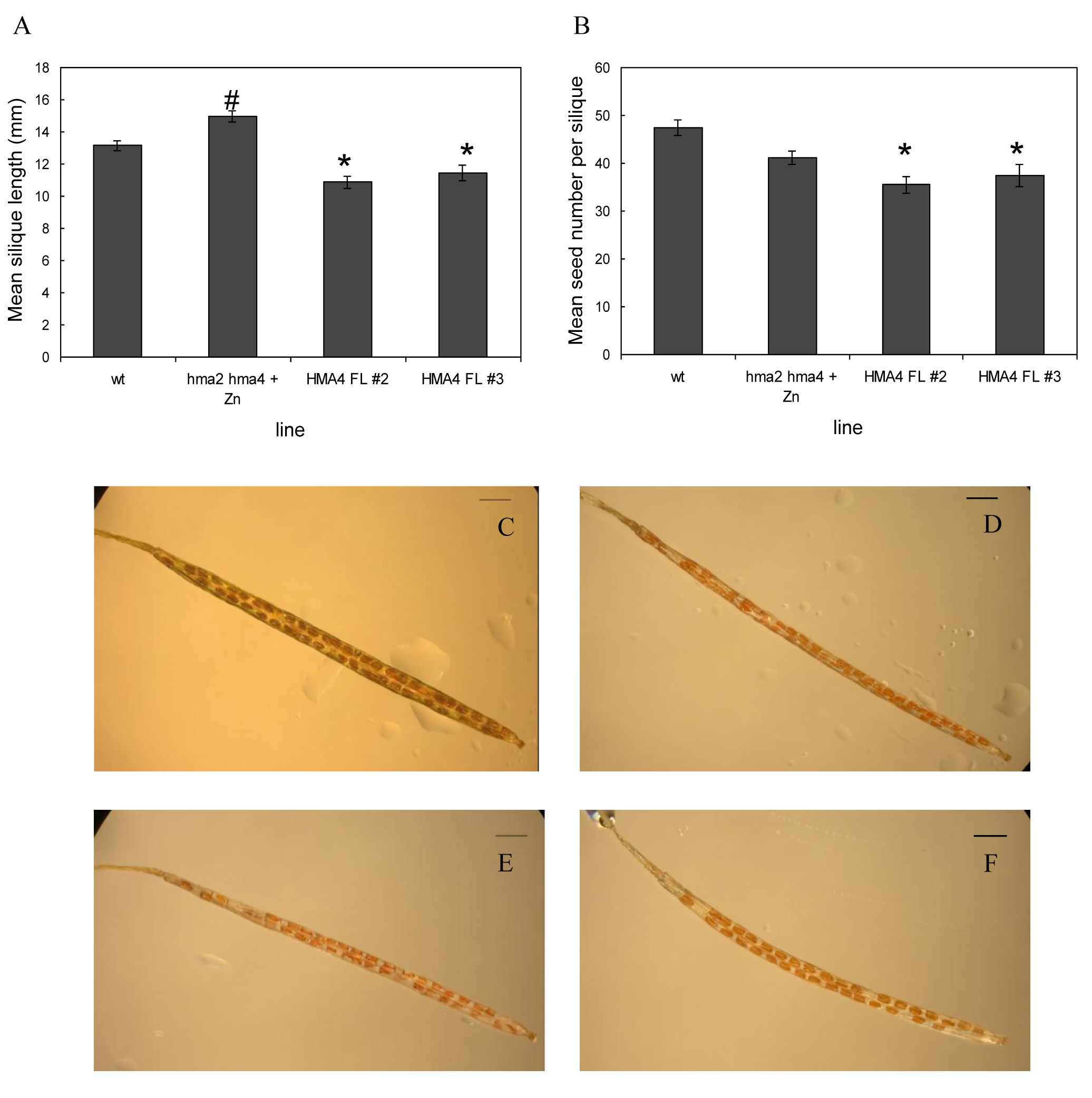

Supplement: Figure S3 — Silique lengths and number of seeds per silique is reduced in the AtHMA4-FL lines. Silique lengths (A) and number of seeds per silique (B) taken from lines after 49 days growth on soil. Plants were watered with tap water apart from the +Zn plants which were watered with 3 mM ZnSO4. Silique lengths are the mean ± S.E. of 60 siliques from six plants while values for seed per silique were determined from 36–40 siliques from five plants. Significant differences are indicated: # significantly greater than wt; * significantly lower than wt (Students t test, P<0.05). Example siliques are shown for wt (C), hma2 hma4 + Zn (D), AtHMA4-FL line 2 and AtHMA4 FL line 3. Scale bar = 1 mm. (0.86 MB TIF) [file pone.0013388.s003.tif]

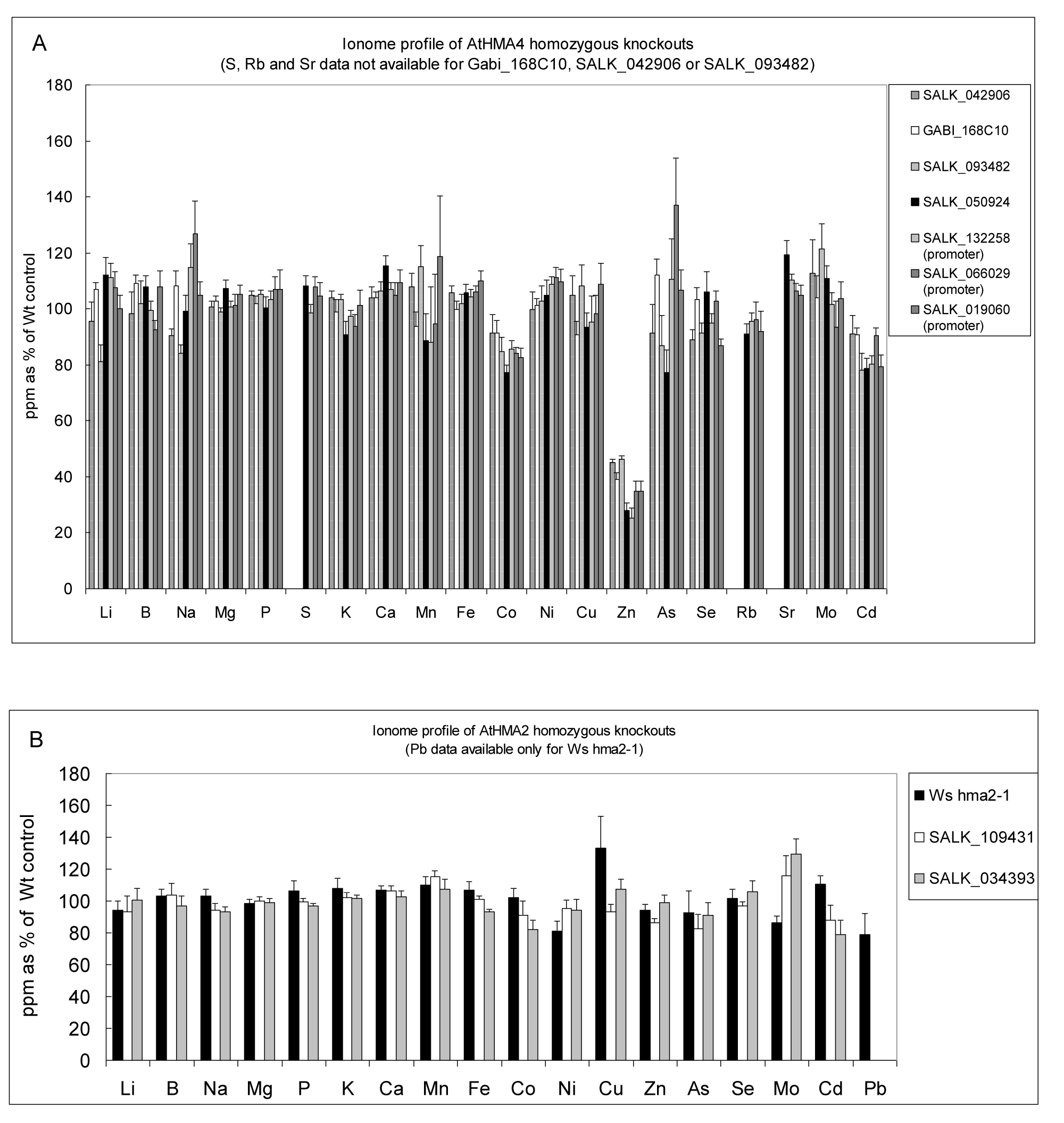

Supplement: Figure S4 — Ionomic profiles for shoots of T-DNA insertion lines. A, hma2 mutants; B, hma4 mutants. Values are the mean +/− S.E. (n = 12) expressed as % of values for wt. (0.66 MB TIF) [file pone.0013388.s004.tif]

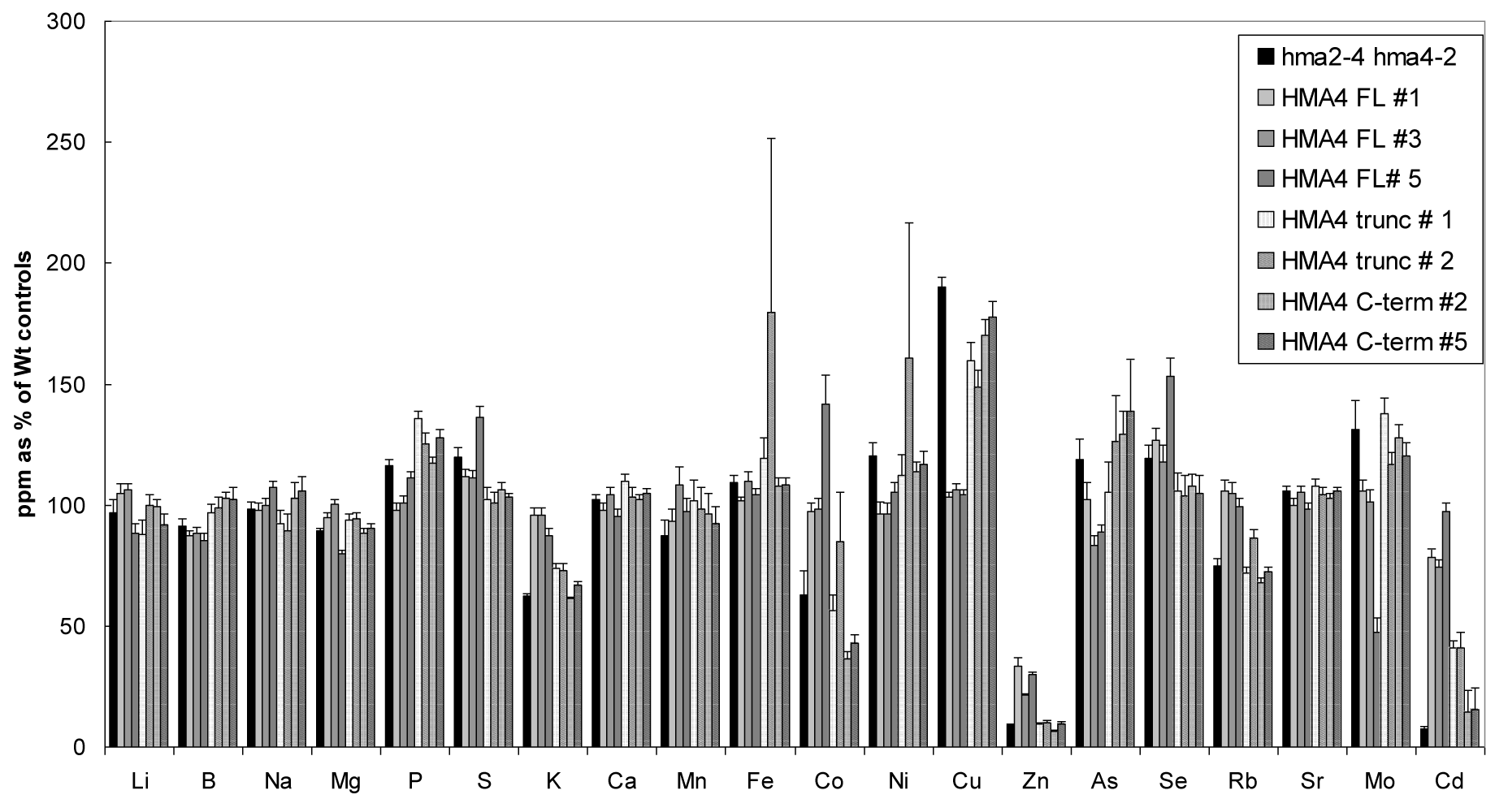

Supplement: Figure S5 — Ionomic profiles for shoots of selected lines of hma2 hma4 expressing either AtHMA4-FL, AtHMA4-trunc or AtHMA4-C-term. Values are the mean +/− S.E. determined from 12 plants expressed as % of values for wt. (0.47 MB TIF) [file pone.0013388.s005.tif]
